# Multidimensional Poverty and Child Survival in India

**DOI:** 10.1371/journal.pone.0026857

**Published:** 2011-10-27

**Authors:** Sanjay K. Mohanty

**Affiliations:** Department of Fertility Studies, International Institute for Population Sciences, Mumbai, Maharashtra, India; Aga Khan University, Pakistan

## Abstract

**Background:**

Though the concept of multidimensional poverty has been acknowledged cutting across the disciplines (among economists, public health professionals, development thinkers, social scientists, policy makers and international organizations) and included in the development agenda, its measurement and application are still limited.

**Objectives and Methodology:**

Using unit data from the National Family and Health Survey 3, India, this paper measures poverty in multidimensional space and examine the linkages of multidimensional poverty with child survival. The multidimensional poverty is measured in the dimension of knowledge, health and wealth and the child survival is measured with respect to infant mortality and under-five mortality. Descriptive statistics, principal component analyses and the life table methods are used in the analyses.

**Results:**

The estimates of multidimensional poverty are robust and the inter-state differentials are large. While infant mortality rate and under-five mortality rate are disproportionately higher among the abject poor compared to the non-poor, there are no significant differences in child survival among educationally, economically and health poor at the national level. State pattern in child survival among the education, economical and health poor are mixed.

**Conclusion:**

Use of multidimensional poverty measures help to identify abject poor who are unlikely to come out of poverty trap. The child survival is significantly lower among abject poor compared to moderate poor and non-poor. We urge to popularize the concept of multiple deprivations in research and program so as to reduce poverty and inequality in the population.

## Introduction

The paper has two empirical goals; i) to measure the state of multidimensional poverty in India and ii) to examine the state of child survival among the abject poor, moderate poor and non-poor households in India. We conceptualized the paper with the following rationale. First, though multidimensional poverty has been acknowledged cutting across the disciplines (among economists, development thinkers, social scientists, public health professionals, policy makers and international organizations) and included in the development agenda, its measurement and application are still limited. Second, poverty eradication program in India identifies poor using the concept of multidimensional poverty but the official estimates of poverty continue to be derived from consumption expenditure data. Third, empirical evidences suggest an inverse association of level and inequality in child survival, that is, as mortality declines, the gap in child mortality between the poor and the better-off widens [1]. Fourth, in transitional economies, health care services are more likely to benefit the non-poor than the poor [2]. Along with these goals and rationale, we hypothesize that there are no significant differences in child survival (infant mortality rate and under-five mortality rate) among the educational poor, wealth poor and health poor in India.

In deriving multidimensional poverty, both theoretical and methodological issues are of immense importance. Methodological issues include the fixing of cut off point for the poor and non-poor, aggregation of multiple dimensions into a single index, weighting of dimensions and the unit of analyses, while theoretical issues relate to the choice of dimensions, choice of indicators and the context [3], [4], [5]. The UNDP has devised two composite indices, namely the Human Poverty Index 1 (HPI 1 for developing countries) and Human Poverty Index 2 (HPI 2 for developed countries) to measure the state of multidimensional poverty in the domain of health, knowledge and living standard [6]. Among researchers, there is general agreement in specifying the poverty line of each dimension, but they differ in deriving the aggregate poverty line. While some have used the union (poor in any dimension) approach [7], others have used the intersection approach (poor in two or more dimension) [8] or relative approach [9] in fixing the poverty line. On the theoretical front, the dimensions of education, health and income are often measured and few studies have included subjective well being such as fear to face hardship in defining multidimensional poverty [10]. Studies also document varying degrees of correlation between dimensions of poverty [11].

Traditionally in money-metric form, poverty estimates were primarily based on income and/or consumption expenditure survey data. Recently data from the Demographic and Health Surveys (DHS) were used in estimating poverty of selected African countries. Along with consumer durables and housing characteristics, the educational level of head of household was used in defining poverty [12], [13]. Using three rounds of Indian DHS data, studies also estimated the change in deprivation level in Indian states [14]. Most recently, using unit data from three large scale population based surveys (DHS, MICS and WHS), the multidimensional poverty index (MPI) was estimated for 104 developing countries [15]. It used a set of 10 indicators in three key dimensions of education, health and living standard, assigned equal weight to each dimension and equal weight to the variables within the dimension. The results were robust, capture the multiple deprivations and disseminated in the 2010 Human Development Report [16]. India ranked 74^th^ among 104 countries with a MPI value of 0.29. Recently, studies elaborated the strengths, limitations, and misunderstanding of multidimensional poverty measurement and viewed that the methodology of MPI satisfies the basic axioms of multidimensional poverty measurement and can be decomposed by population sub-groups and dimensions [3].

In India, the estimates of poverty and the identification of poor for conditional cash transfer are carried out independently. The official estimates of poverty are derived by the Planning Commission based on consumption expenditure data collected by the National Sample Survey Organization (NSSO) in its quinquinneal round (since 1973–74). On the other hand, the poor are identified by a Below Poverty Line (BPL) Survey carried out by the District Rural Development Authority (DRDA) of each state with guidelines from the Ministry of Rural Development, Government of India. Based on the Planning Commission, Government of India estimates of 2004–05 (uniform recall period), 27% of India's population (25.7% urban and 28.3% rural) were living below the poverty line [17]. However, these estimates are often debated and revised owing to different recall periods (365 vs. 30 vs. 7 days) in various rounds, the fixed basket of goods and services, the price index applied and appropriate minimum threshold. Additionally, the consumption expenditure is sensitive to household size and composition and the official poverty estimates in India are not adjusted for household size and composition. Recently, the Government of India appointed the Tendulkar Committee to suggest an amendment of poverty estimates. The Committee recommended the same poverty estimates for urban India (25.7%) but re-estimated rural poverty for 2004–05 [18]. On the other hand, three rounds of BPL survey had already been carried out with different methodology for identifying the poor. The first BPL survey was conducted in 1992, the second in 1997 and the third in 2002. There were improvements in the methodology in successive rounds of BPL surveys but all these rounds used the concept of multidimensional poverty. For example, the 2002 round used a set of 13 socioeconomic indicators (size of operational land holding, type of house, availability of food and clothing, security, sanitation, ownership of consumer durables, literacy status, status of household labour, means of livelihood, status of school going children, type of indebtedness, reason for migration and preference of assistance) with a score ranging from 0 to 4 for the variables. The total score ranged from 0 to 52 and the states were given the flexibility of deciding the cut off points. There has been discontent on the methodology used in BPL surveys, misuse in the distribution of BPL cards [19], [20] and researchers have suggested methodological improvements in determining the BPL status [21], [22].

Evidences in India suggest reduction in consumption poverty, but the state of child health has not improved substantially. During 1992–2006, the proportion of undernourished children had declined marginally (about two-fifths of children were undernourished in 2005–06). The infant mortality rate had declined from 77 per 1000 live births in 1991–95 to 57 per 1000 live births in 2001–05 [23]. Though there is large differential in the state of child health and maternal care utilization by education and wealth status of the households [24]–[26] little is known on the state of child health by multiple deprivations. This paper attempts to measure the deprivation in multiple dimensions of capability and understand its linkage with child survival in India, using large scale population based survey data. We have used the word deprivation and poverty interchangeably.

## Materials and Methods

In the last two decades, the Demographic and Health Surveys (DHS) have bridged the data gap on population, health and nutrition parameters of many developing countries, including India. The DHS in India, known as the National and Family and Health Survey (NFHS), was first conducted in 1992–93 and the second and the third rounds were conducted in 1998–99 and 2005–06 respectively. The NFHSs are large scale population based representative sample surveys that cover more than 99% of India's population under rigorous conditions of scientific sampling design, training of investigators and high quality data collection and edit procedures. These surveys collect reliable information on births, deaths, family planning, nutrition, a range of health related issues including HIV/AIDS and the living conditions of households. There were improvements in coverage and dimensions in successive rounds of the survey.

NFHS 3 canvassed three different survey instruments namely, the household schedule, the women's questionnaire and the men's questionnaire from the sampled households. The household schedule collected information on economic proxies such as housing quality, household amenities, size of land holding and consumer durables, whereas the women questionnaire collected detailed information on reproductive histories, health, nutrition and related information of mothers and children. The women's questionnaire also recorded the detailed birth history from sampled women that provides an opportunity to estimate the infant mortality and under-five mortality rates. The men's questionnaire collected information on men's involvement in health care, reproductive intention and knowledge and use of contraception from men in the age group 15–54. A detailed description of the survey design of the NFHS and the findings are available in the national report [23]. In this paper we have utilized the data of NFHS 3 that covered a sample of 109,041 households and 124,385 women in the country [Appendix S1]. The household file, women's file, birth history file and the member files are used in the analysis.

We have measured multidimensional poverty in the dimension of education, health and living standard of the household. The dimension of education includes literacy status of all adult members and the current schooling status of school going children in the households. The living standard is measured by a set of economic proxies of the household. The dimension of health includes child nutrition and the health of women in the age group 15–49. In deriving the estimate of multidimensional poverty, the unit of analysis is the household, whereas the child is the unit of analysis for child health variables. Bi-variate analysis is used in understanding the differentials in poverty while the principal component analysis (PCA) is used in estimating the wealth index. The estimates of IMR and U5MR are derived from the birth history file and analyses were carried out separately for rural and urban areas. The life table technique is used to estimate the IMR (probability of dying in first year of life) and the U5MR (the probability of dying within first five years of life) by poverty level of the household. The SPSS 14 and STATA 10 software packages are used.

## Results

Results are presented in two sections. Section 1 describes the methodology of identification of poor and estimates of multidimensional poverty and section 2 describes child survival among the abject poor, moderate poor and non-poor.

### Identification of the Poor and the Extent of Multidimensional Poverty

Table 1 reports the specific indicators used in quantifying dimensional poverty in education, health and living standard separately for rural and urban areas. It also provides the method of fixing the cutoff point of poor in each of these dimensions.

**Table 1:** Dimensional indicators of poverty and the method of deriving poor in India.

Dimension	Indicators for Rural	Indicators for Urban	Defining Poor
**Education**	No adult literate member in the household	No adult literate member in household	Household do not have an adult literate member or any of the child age 7–14 in the household never attended or discontinued school
	Any child in the school going age (7–14) never attended school	Any child in the school going age (7–14) never attended school	
	Any child in the school going age (7–14) discontinued schooling	Any child in the school going age (7–14) discontinued schooling	
**Health**	Any child below 5 years of age is severely underweight	Any child below 5 years of age is severely underweight	Either any child in the household is severely underweight or any woman is severely/moderately anemic
	Any woman age 15–49 years is severely or moderately anaemic	Any woman age 15–49 years is severely or moderately anaemic	
**Wealth**	**Housing Condition:**	**Housing Condition:**	Derived from the composite wealth index using the PCA.
	Floor type, wall type, roof type, window type	Floor type, wall type, roof type, window type,	The cut off point of poor in is 26% in urban areas and 28% in rural areas. This cut-off point is equivalent to the poverty estimates of the Planning Commission, Govt. of India, 2004–05
	Persons per room	Persons per room, own house	
	Access to improved water	Access to improved water	
	Type of cooking fuel	Type of toilet facility	
	Electricity	Type of cooking fuel	
	Separate kitchen	Separate kitchen	
	**Consumer Durables:**	**Consumer Durables:**	
	Motorcycle, car, landline telephone, mobile, television, pressure cooker, refrigerator, computer, sewing machine, watch, bicycle, radio	Motorcycle, car, landline telephone, mobile, television, pressure cooker, refrigerator, computer sewing machine, watch	
	**Size of Landholding:**		
	No land, marginal, small, medium/large holdings		
	**Agricultural accessories:**		
	Thresher, Tractor, Water Pump		

We define a household as poor in the education domain, if the household does not have a single literate adult (15+ years, as used in India) or if any children in school going age (7–14 years) are out of school because they have never enrolled or discontinued schooling. The literacy status of any adult member in a household is the basic and frequently used indicator that measures progress in educational development in India. It is computed by the presence or absence of any adult literate member in the household. We prefer to use this indicator to that of the head of household as the average age of the household head is 46 years in the country. In such cases, the recent benefits of education (say in last 10–15 years) to the members of household will not be captured, while the educational level of any adult member will capture such changes. Second, the official age of child schooling in India is 6–14 years but we prefer to use the age group 7–14 years because the survey was conducted during November 2005-August 2006 and the child's age was estimated as of the survey date. It was found that 20% of the households did not had an adult literate member, 9% of the households had at least one child who had never gone to school and 4.8% households had at least one child who had discontinued schooling (Table 2).

**Table 2:** Mean and confidence interval of dimensional indicators of education and health by place of residence in India, 2005–06.

Dimensional Indicators	Combined		Rural		Urban	
	Mean	95% CI	Mean	95% CI	Mean	95% CI
**Education**						
Households without a single adult literate member	0.198	0.196–0.201	0.253	0.249–0.256	0.853	0.083–0.088
Household with at least one child (7–14 years) who has never gone to school	0.085	0.083–0.086	0.104	0.102–0.107	0.044	0.042–0.046
Household with at least one child (7–14 years) who has discontinued schooling	0.048	0.047–0.050	0.054	0.052–0.056	0.035	0.033–0.036
**Health**						
Household with at least one women aged 15–49 years who is severely/moderately anaemic	0.164	0.162–0.166	0.176	0.173–0.179	0.14	0.137–0.143
Household with at least one child aged 0–59 months who is severely underweight	0.058	0.056–059	0.071	0.069–0.730	0.03	0.029–0.032

The NFHS 3 had collected information on self reported health and biomarkers from women aged 15–49, men aged 15–54 and children under five years of age to assess the health condition of the population. The biomarkers include the measurement of height and weight, measuring anaemia level and HIV testing in sub-section of the sampled population. These variables are further used in deriving the nutritional measures of children (height for age, weight-for age and weight-for height), nutritional measures men and women (Body Mass Index), the anemia levels (mild, moderate, severe and not anemic) and the HIV prevalence. Among these indicators we prefer to use the weight-for age that reflects both the acute and chronic under-nutrition of children and the anemia of women to quantify the health dimension of the population as these indicators are widely recognized health measures for children and mothers. The selections of these two indicators are also guided by the following consideration. First, undernutrition of children and maternal health are priority agenda in India's health program [27]. Second, undernutrition among children is the leading cause of under-five mortality in developing countries including India and linked to cognitive development of children [28]–[31]. Also, child health and child development are intertwined. Third, anaemia is one of the risk factor of maternal mortality and morbidity and determinant of child health [32], [31]. Finally, among other factors, the programmatic intervention can significantly reduce the under-nutrition of children and anaemia of the women and hence can alleviate health poverty [33].

However, as 43% children under age five are underweight and 55% women are anaemic (either moderate or mild or severe) in the country, we prefer to use the severity in these parameters in defining the health poor. We consider a household poor in the health domain if the household has at least a child who is severely underweight (children whose weight for age is below minus three standard deviation from the median of the reference population) or a woman who is severely or moderately anaemic (hemoglobin level less than 9.9 g/dl). It may be mentioned that information on blood sample was not collected in the state of Nagaland and so the variable for the state is not used. We prefer the anemia to BMI as the measurement of BMI excludes pregnant women and women who had given birth in two months preceding the survey, are age sensitive where as anaemia is of program priority.

In the wealth domain, economic proxies (housing conditions, household amenities, consumer durables, size of land holding) of the household are used in explaining the economic differentials in population and health parameters as DHS does not collect data on income or consumption expenditure. These economic proxies are combined to form a composite index, often referred to as the wealth index and the PCA is the most frequently used method in deriving the wealth index. The utility of wealth index in explaining economic differentials in population and health parameters have been established [34]–[35]. However, our wealth index differs from the DHS wealth index in many aspects. First, we have constructed the wealth indices for rural and urban areas separately using the PCA, as estimates of health care utilization differ significantly when separate wealth indices are used for rural and urban areas rather than a single index [36]. Second, we have carefully selected variables based on theoretical and statistical significance in the construction of the wealth index for rural and urban areas. For example, the DHS wealth index does not include land in the construction of the wealth index, but uses agricultural accessories such as tractors and threshers. We have used agricultural related variables for rural but not for urban areas. Similarly, in rural areas a large proportion of households own a house, therefore we have not included this variable in the construction of the wealth index for rural India. Third, we have equated the cut-off point of the poor to the Planning Commission, Government of India estimates of poverty in 2004–05, based on uniform recall period. Accordingly, 26% of urban households and 28% of rural households were considered poor in the economic domain. We are aware that the distribution of asset and consumption may not have one to one correspondence. However, the correlation coefficient of percentage of population living below poverty line based on calories intake (also referred as consumption poverty) and the percentage of population in first wealth quintile (as defined in NFHS 3 data) was 0.8 (state level). The mean, 95% confidence interval and the factor score (weight) of the variables used in deriving wealth indices are shown in Table 3.

**Table 3:** Mean, 95% confidence interval and factor score of variables used in the construction of wealth index by place of residence, India, 2005–06.

	Rural			Urban		
Variables	Mean	95% CI	Factor score	Mean	95% CI	Factor score
**Housing quality**						
Floor type	0.305	0.301–0.309	0.253	0.807	0.803–0.810	0.212
Wall type	0.533	0.530–0.538	0.237	0.889	0.886–0.892	0.204
Roof type	0.714	0.711–0.718	0.165	0.924	0.922–0.927	0.166
No window	0.412	0.408–0.416	−0.239	0.151	0.148–0.154	−0.216
Window without cover	0.290	0.286–0.293	0.022	0.216	0.212–0.219	−0.109
Window with cover	0.299	0.295–0.303	0.235	0.633	0.629–0.638	0.253
**Person per room**						
Two person	0.325	0.321–0.328	0.056	0.376	0.372–0.381	0.093
2–4	0.426	0.422–0.430	0.026	0.431	0.426–0.435	−0.002
4+	0.249	0.246–0.253	−0.090	0.193	0.190–0.197	−0.111
Own house	0.933	0.931–0.935	***	0.782	0.779–0.786	0.042
Improved drinking water	0.848	0.846–0.851	0.048	0.960	0.958–0.962	0.038
Cooking fuel	0.088	0.086–0.090	0.233	0.601	0.597–0.606	0.285
Electricity	0.558	0.553–0.561	0.229	0.931	0.928–0.933	***
Separate kitchen	0.440	0.436–0.444	0.173	0.634	0.630–0.638	0.241
**Toilet facility**						
No toilet	0.740	0.737–0.744	***	0.169	0.165–0.172	−0.247
Pit toilet	0.060	0.058–0.062	***	0.044	0.042–0.046	−0.058
Flush toilet	0.200	0.197–0.203	***	0.787	0.0784–0.791	0.255
**Consumer durables**						
Pressure cooker	0.221	0.218–0.225	0.283	0.699	0.695–0.703	0.266
Television	0.301	0.298–0.305	0.281	0.732	0.728–0.735	0.237
Sewing machine	0.126	0.124–0.129	0.209	0.309	0.305–0.313	0.178
Mobile	0.074	0.072–0.0757	0.227	0.363	0.359–0.368	0.243
Telephone	0.080	0.078–0.0819	0.244	0.266	0.263–0.271	0.239
Computer	0.006	0.005–0.006	0.093	0.080	0.078–0.083	0.157
Refrigerator	0.066	0.064–0.068	0.230	0.334	0.331–0.339	0.271
Watch	0.714	0.710–0.718	0.192	0.911	0.908–0.913	0.152
Motorcycle	0.108	0.106–0.111	0.245	0.305	0.301–0.309	0.232
Car	0.010	0.009–0.011	0.122	0.061	0.059–0.063	0.145
Radio	0.270	0.0267–0.274	0.161	0.389	0.385–0.393	***
Bicycle	0.517	0.512–0.520	0.083	0.501	0.497–0.506	***
**Land and agricultural accessories**						
No land	0.415	0.411–0.419	−0.057	0.810	0.806–0.813	***
Marginal holding (up to 2.5 acres)	0.392	0.389–0.396	−0.036	0.111	0.108–0.113	***
Small holding (2.51–5)	0.082	0.080–0.084	0.111	0.038	0.036–0.040	***
Medium/large (5+)	0.110	0.108–0.113	0.048	0.041	0.040–0.043	***
Irrigated land	0.381	0.377–0.385	0.080	0.125	0.123–0.128	***
Water pump	0.099	0.096–0.101	0.150	0.110	0.107–0.113	***
Threshers	0.022	0.021–0.023	0.082	0.004	0.004–0.005	***
Tractors	0.023	0.022–0.024	0.121	0.005	0.004–0.005	***

***Not used in the analyses.

The weight of the variables generated in the construction of wealth indices are in the expected direction, both in urban and rural areas. The variables that reflect a higher standard of living have a positive weight, while those with a lower standard of living have a negative weight. For example, the weight of a flush toilet in urban areas is 0.255, pit toilet is −0.058 and that of no toilet is −0.247. The distribution of the wealth index showed that it is positively skewed in urban areas and negatively skewed in rural areas. The alpha value is 0.86 in urban and 0.81 in rural areas indicating that the estimates are reliable. Based on the ascending order of the composite index, a percentile distribution is obtained for the household both in rural and in urban areas.

Based on dimensional scores, we have classified a household as abject poor if it is poor in at least two of the three dimensions, moderate poor if it is poor in only one dimension and non-poor if it is not poor in any one of the dimensions (Table 4). Similarly, a household is classified as poor, if it is poor in at least one dimension. Results indicate that 27% of the households in India are poor in education and wealth dimensions each, while 21% are poor in the health dimension. The distribution of households in multidimensional poverty score suggests that 31% of the households in India are poor in one dimension, 17% are poor in two dimensions, 4% are poor in all three dimensions and 48% are non-poor. Based on the classification, 20% of the households in the country are said to be abject poor and 52% poor (inclusive of abject poor) with large rural-urban differentials.

**Table 4:** Percentage of poor in dimension of education, health and wealth and the overall poverty in India, 2005–06.

Poverty levels of Households	Combined	Rural	Urban
Percentage of households poor in education	27.3	33.7	14.1
Percentage of households poor in health	20.6	22.7	16.3
Percentage of households poor in wealth	27.0	28.0	26.0
**Overall Poverty status**			
Percentage of non-poor households	48.3	43.2	58.9
Percentage of households poor in one dimension	31.6	33.4	27.7
Percentage of households poor in two dimensions	16.5	19.1	11.3
Percentage of households poor in all three dimensions	3.6	4.3	2.1
Total Percent	100	100	100
**Classification of poverty**			
Percentage of Non-poor households	48.3	43.2	58.9
Percentage of households Abject poor (Poor in at least two or more dimensions)	20.1	23.4	13.3
Percentage of households Poor (Including abject poor)	51.7	56.8	41.1

The classification of households on economic, education and health dimensions suggests that those who are economically poor are more likely to be educationally poor both in rural and urban areas. Among those economically poor, about half of them are educationally poor compared to one-sixth among the economically non-poor. However, the differentials in economically poor and health poor are not large.

We further validated the multidimensional poverty estimates with three critical variables; namely household with a BPL card, an account in a bank or post office and coverage under the health insurance scheme (Table 5). The possession of a BPL card entitled a household to take benefits from the various poverty eradication schemes of the national and state governments such as subsidized ration, guaranteed employment, free housing and maternal benefits etc. A higher proportion of abject poor households possess a BPL card compared to the moderate poor or non-poor households. However, it also indicates that the majority of poor households are not covered under the poverty eradication program. Similarly, 14% of abject poor households had a bank or a post office account compared to 33% among the moderate poor and 55% among non-poor indicating the limited access of abject poor and poor to financial institutions. The coverage of health insurance in the population is low and almost non-existent among abject poor. These classifications also validate the measure of multidimensional poverty and suggest that the poor are disadvantaged in the service coverage.

**Table 5:** Percentage of households covered under BPL scheme, access to financial institution, covered under health insurance and living in slums by poverty levels in India, 2005–06.

	Abject Poor	Moderate Poor	Non-poor	All
**Combined**				
Households have a BPL card	37.3	31.3	20.6	27.3
Households have an account in a bank or post office	14.3	33.1	55.1	40.2
Any adult member in the household covered under a health insurance scheme	0.6	2.9	8.2	5.0
**Rural**				
Households have a BPL card	39.1	35.6	27.5	32.9
Households have an account in a bank or post office	12.5	28.9	45.6	32.3
Any adult member in the household covered under a health insurance scheme	0.2	1.6	4.0	2.3
**Urban**				
Households have a BPL card	30.7	20.7	10.2	15.9
Households have an account in a bank or post office	20.9	43.5	70.8	56.5
Any adult member in the household covered under a health insurance scheme	2.1	6.3	14.8	10.7
Lives in a slum	59.6	50.4	31.7	37.3

**Figure 1:**
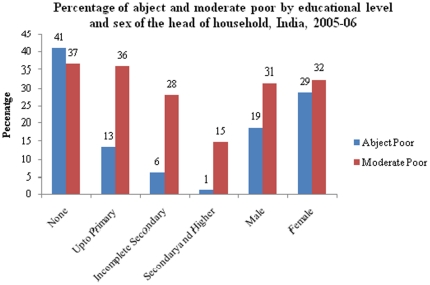
Percentage of abject poor and moderate poor by educational level and sex of the head of the household, India, 2005–06. X axis: Educational Attainment Y axis: Percentage abject poor/moderate poor. Red Bar: Moderate poor, Blue Bar: Abject Poor.

Prior research suggests that the extent of multidimensional poverty is higher among female headed households, household heads with low educational level and among large households [37], [9]. We have examined the differentials in multidimensional poverty by the selected characteristics of the head of the household such as age, sex, educational level, marital status and household size and found a similar pattern (Figure 1). In general, it is observed that the extent of abject poverty and moderate poverty decreases with age (result not shown), educational level of households, households with many members and that it is higher among female headed households.

Given the demographic and developmental diversity in the country, we have estimated the extent of multidimensional poverty in the states of India and compared it with consumption poverty estimates based on uniform recall period by the Planning Commission, Government of India for the period 2004–05 (Table 6). Based on the estimates of abject poverty, we have classified the states of India as follows.

**Table 6:** Percentage of abject poor households, moderate poor households and the percentage of population living below the poverty line (consumption poverty) in the states of India, 2005–06.

Sr No	States	Combined		Rural		Urban		Percentage of population living below poverty line, 2004–05		
		Abject poor	Moderate poor	Abject poor	Moderate poor	Abject poor	Moderate poor	Combined	Rural	Urban
1	Kerala	1.2	14	1	13.5	1.6	15.1	15	13.2	20.2
2	Himachal Pradesh	1.8	22	1.7	22.7	2.6	16.2	10	10.7	3.4
3	Goa	4.5	18.5	3.5	16.3	5.2	20.2	13.8	5.4	21.3
4	Delhi	5.5	19.6	0.9	24.8	5.9	19.2	14.7	6.9	15.2
5	Punjab	5.7	28	4.7	30.7	7.1	23.9	8.4	9.1	7.1
6	Sikkim	5.7	31	6.1	32.8	4.1	23.8	20.1	22.3	3.3
7	Mizoram	6.1	20.3	7.9	23.3	4.6	17.8	12.6	22.3	3.3
8	Jammu and Kashmir	7.2	30.9	8.1	34.2	5.4	23.5	5.4	4.6	7.9
9	Manipur	8.3	26.7	7.9	23.1	9.3	34.4	17.3	22.3	3.3
10	Uttaranchal	8.5	26.5	8.3	28.5	8.7	21.4	39.6	40.8	36.5
11	Haryana	10.1	31.3	10.1	33.9	10.1	25.6	14	13.6	15.1
12	Maharashtra	11.2	28.7	15	32.5	7.2	24.6	30.7	29.6	32.2
13	Nagaland	11.7	28.4	12.3	29.1	10.1	26.6	19	22.3	3.3
14	Karnataka	11.8	32	12.4	35.3	10.8	27.1	25	20.8	32.6
15	Gujarat	12.5	33.7	14.1	36.5	10.4	29.8	16.8	19.1	13
16	Tamil Nadu	13.4	32	11.8	33.2	15.2	30.6	22.5	22.8	22.2
17	Tripura	13.7	28.9	12.4	27.2	19.6	37.1	18.9	22.3	3.3
18	Andhra Pradesh	19.6	35.9	19.1	37.2	20.6	32.9	15.8	11.2	28
**19**	West Bengal	20.4	30.4	24.4	32.2	12.1	26.6	24.7	28.6	14.8
20	Meghalaya	21.8	34.8	25.9	37.5	10.3	27.2	18.5	22.3	3.3
**21**	Assam	23.1	36	25.7	35.1	12.8	39.5	19.7	22.3	3.3
22	Chhattisgarh	24.9	35	27.2	35.2	16.6	34.4	40.9	40.8	41.2
**23**	Uttar Pradesh	24.9	33.6	27	35.6	18.5	27.8	32.8	33.4	30.6
24	Rajasthan	25.4	34.2	30.7	36.5	12.5	28.5	22.1	18.7	32.9
**25**	Arunachal Pradesh	25.9	26.1	27.7	35.3	21.4	38.1	17.6	22.3	3.3
26	Orissa	28.3	32.1	30	32	20	33	46.4	46.8	44.3
**27**	Madhya Pradesh	30.3	32.7	34.9	33.7	18.6	30.2	38.3	36.9	42.1
28	Jharkhand	37.8	31.8	45	32.4	16.6	30.1	40.3	46.3	20.2
**29**	Bihar	39.4	31.4	41.5	32	28.3	28.3	41.4	42.1	34.6
	**India**	20.1	31.6	23.4	33.4	13.3	27.7	27.5	28.3	25.7
	SD (All states)	10.6	5.6	12.3	6.1	6.6	6.3	11.2	12.1	14.1
	Mean	15.9	29.2	17.1	30.8	11.9	27.4	22.8	23.4	18.7
	CV	66.5	19.2	72.0	19.9	55.0	23.2	49.0	51.6	75.4

States with abject poverty of more than 20%: Bihar, Jharkhand, Madhya Pradesh, Orissa, Arunachal Pradesh, Rajasthan, Uttar Pradesh, Chhattisgarh, Assam, Meghalaya and West Bengal.

States with abject poverty of 10%–20%: Andhra Pradesh, Tripura, Tamil Nadu, Gujarat, Karnataka, Nagaland, Maharashtra and Haryana.

States with abject poverty of less than 10%: Uttaranchal, Manipur, Jammu and Kashmir, Mizoram, Sikkim, Punjab, New Delhi, Goa, Himachal Pradesh and Kerala.

Among all the states of India, the extent of abject poverty and the overall poverty is maximum in the state of Bihar followed by Jharkhand and least in the states of Kerala followed by Himachal Pradesh and Goa. It is observed that the states where the extent of abject poverty is high, the overall poverty is also high. Further, the pattern of multidimensional poverty generally follows the state of human development in these states. For example, the states such as Kerala, Tamil Nadu, Goa, and Himachal Pradesh with higher ranking in human development index [38] have higher rank in multidimensional poverty and the states such as Bihar, Uttar Pradesh, and Madhya Pradesh with lower rank in HDI also have lower rank in multidimensional poverty. The inter-state differentials in abject poor are large compared to moderate poor, both in rural and urban areas. The coefficient of variation of abject poor in states of India is 67 compared to 19 for moderate poor (combined).

We have attempted to understand the association of dimensional poor and the consumption poor in the states of India. The rank order correlation of wealth poor and education poor (0.78) is higher than that of wealth poor and health poor (0.58). However, the correlation of consumption poor and wealth poor are large and significant (0.70).

### Poverty and Child Survival

Evidence across developing countries suggests substantial reduction in infant and child mortality in the last two decades. While immunization of children was primarily attributed in improving child survival in the 1980s, reduction in poverty and malnutrition, improvement in the environmental conditions, the use of health services by the mother were significant factors in the reduction of infant and child mortality in the 1990s [39], [40]. In Indian context, the policy guidelines aimed to reduce child mortality from 123 to 41 per 1000 live births by 2015 [41] but improvement in the under-five mortality rate is slow and it accounts for one-fifth of the global under-five mortality rate [42]. Moreover, the health care services in India, like those in other transitional economies, benefit the non-poor more than the poor.

In this section, we discuss the differentials in infant mortality rate and the under-five mortality rate by poverty level in India and the states. The IMR and under-five mortality rate are also two of the 48 monitoring indicators of the millennium development goals and are directly linked to the state of poverty of the households. We have estimated the IMR and U5MR from the birth history file. The reference period in estimating IMR is five years, while it is ten years for U5MR. We have used the life table method in estimating these mortality indicators. Our findings also reveal that the infant mortality rate and the under-five mortality rate are the highest among the abject poor followed by the moderate poor and non-poor both in rural and urban areas. The estimated IMR for India was 64 (95% CI: 60–69) per 1000 live births among the abject poor, 57 (95% CI: 54–61) among the moderate poor and 40 (95% CI: 38–43) among the non-poor (Table 7). Similar differences are found in rural and urban areas.

**Table 7:** Estimated infant mortality rate and under-five mortality rate for five-year periods preceding the survey by place of residence and poverty level, India, 2005–06.

Poverty	Combined				Rural				Urban			
	IMR	95% CI	U5MR	95% CI	IMR	95% CI	U5MR	95% CI	IMR	95% CI	U5MR	95% CI
Over all poverty status												
Non-poor	40	38–43	53	51–55	48	45–52	64	61–67	31	27–34	39	36–41
Moderate poor	57	54–61	78	75–81	60	56–65	85	81–89	53	47–58	65	61–70
Abject poor	64	60–69	103	99–107	67	62–73	110	106–115	57	49–65	84	78–91
Poor including abject poor	60	57–63	88	86–91	63	60–67	99	93–99	54	50–59	72	69–76
**All**	**52**	**50–54**	**73**	**72–75**	**57**	**55–60**	**84**	**82–86**	**42**	**40–45**	**56**	54–58
**Health Dimension**												
Health poor	56	53–60	88	84–91	60	56–64	95	91–99	49	43–55	70	65–76
Health non-poor	49	47–52	67	66–69	56	53–59	78	76–81	40	37–44	52	49–54
**Education Dimension**												
Educationally poor	64	60–68	95	92–99	65	60–70	100	97–104	60	52–69	80	74–86
Educationally Non-poor	47	45–50	64	62–65	54	51–57	74	72–77	39	36–42	49	47–52
**Wealth Dimension**												
Wealth poor	64	60–68	99	96–103	69	63–74	112	107–116	57	52–64	81	76–86
Wealth Non-poor	47	45–49	63	61–64	53	50–56	73	71–76	36	33–39	45	43–48

The estimated under-five mortality rate was 103 (95% CI: 99–107) among the abject poor, 78 (95% CI: 75–81) among moderate poor and 53 (95% CI: 51–55) among the non-poor. The IMR and under-five mortality were higher in rural areas compared to urban areas. The relative standard error of estimated IMR varies in a lower ranges (2–4%) indicating the reliability and statistical significance of the estimates.

Table 7 also provides estimated IMR and U5MR among dimensional poor and nonpoor in India. It is found that the estimated IMR and U5MR are substantially higher among abject poor compared to poor and non-poor in each of the dimension (education, health and wealth). For example, the estimated IMR was 64 (95% CI: 60–68) per 1000 live births among educationally poor compared to 47 (95% CI: 45–50) per 1000 live births among educationally non-poor. We also observed that there are no significant differences in IMR and U5MR with respect to the wealth poor and the education poor at the national level. However, the estimates are marginally lower among the health poor compared to the wealth poor or education poor. For example, the estimated IMR among the education and wealth poor households was 64 each per 1000 live births and 56 among the health poor.

**Figure 2:**
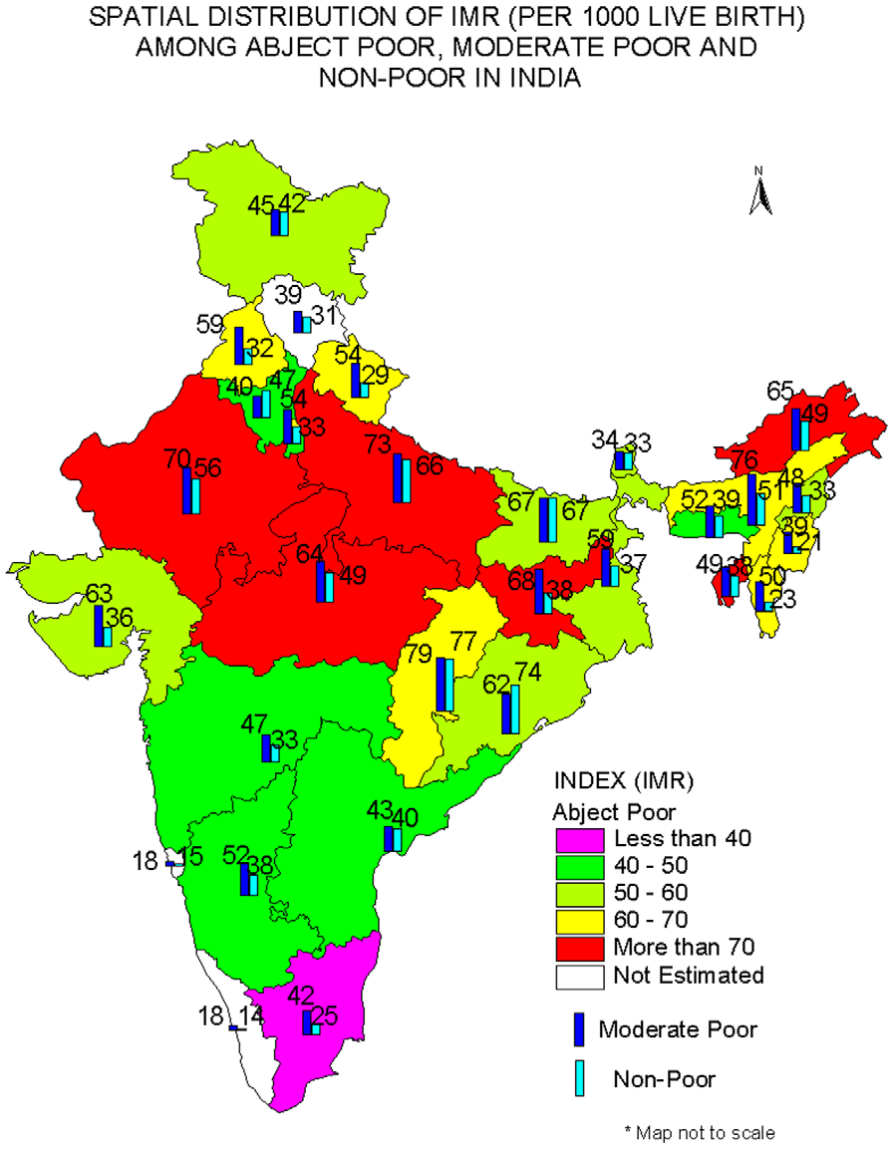
Spatial Distribution of IMR (Per 1000 live births) among abject poor, moderate poor and non-poor in India. Abject poor: Less than 40: Pink color, 40–50: Green, 50–60: Light Green, 60–70: Yellow, More than 70: Red, Blank: Not estimated. Moderate Poor: Blue. Non-poor: Green.

**Figure 3:**
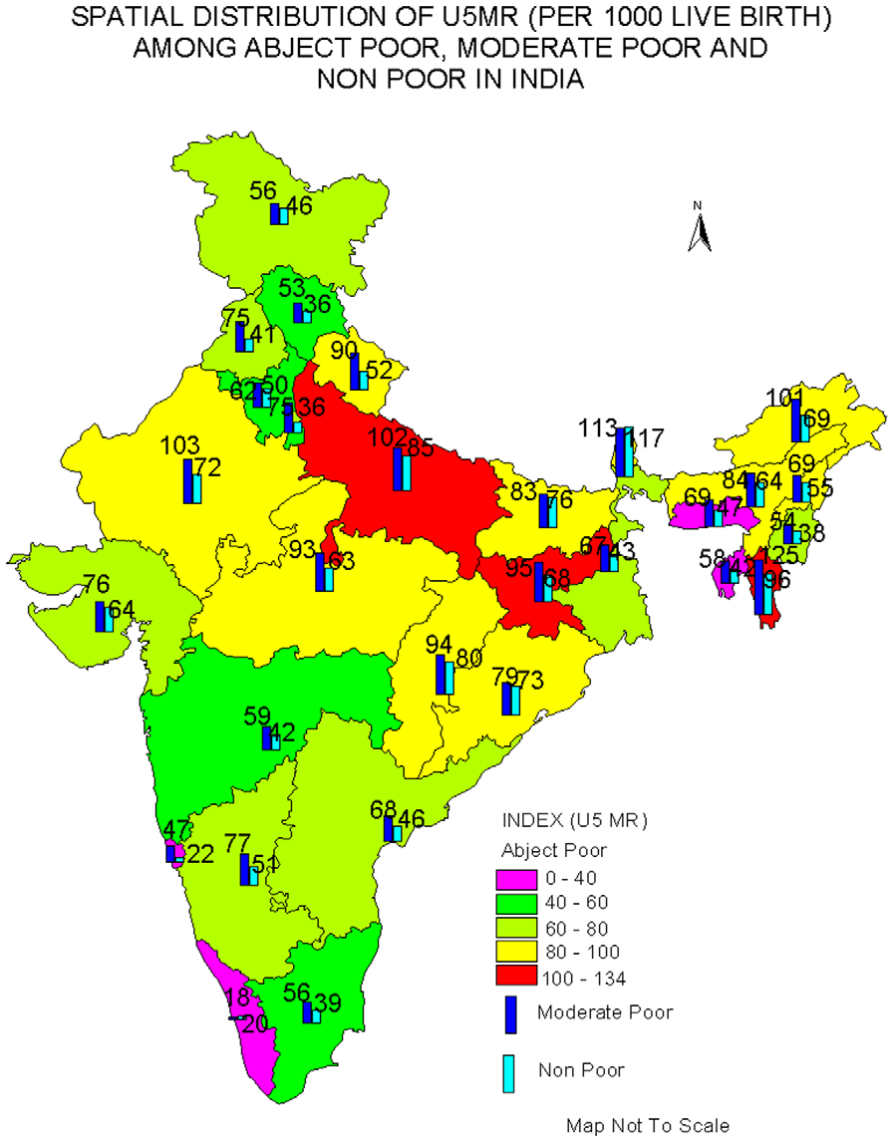
Spatial Distribution of U5MR (Per 1000 live births) among abject poor, moderate poor and non-poor in India. Abject poor: Less than 40: Pink color, 40–60: Green, 61–80: Light Green, 81–100: Yellow,100–134: Red, Moderate Poor: Blue, Non-poor: Green.

The estimated IMR and the under-five mortality rate for the states of India are shown in Figure 2 and Figure 3. In general, the estimated IMR and underfive mortality rate follows a pattern similar to that of the national average; it is highest among the abject poor followed by moderate poor, and least among the non-poor. For example, the estimated IMR among the abject poor in Jharkhand was 83 per 1000 live births compared to 68 among the moderate poor and 38 among the non-poor. Similarly in Uttar Pradesh, the estimated IMR was 82 per 1000 live births among the abject poor compared to 73 among the moderate poor and 66 among the non-poor. Appendix S2 provides the 95% CI of estimated IMR and U5MR for the states of India by abject poor, moderate poor and non-poor. It may be mentioned that for smaller states of India, the CI is large; due to lower sample size.

For comparative purposes, we have classified the states on the basis of differences of IMR among the abject poor and the non-poor. We found that there are nine states, namely, Arunachal Pradesh, Jharkhand, Tripura, Mizoram, Manipur, Punjab, Uttaranchal, Madhya Pradesh and New Delhi, where the differences are more than 25 points. There are ten mores states (Uttar Pradesh, Rajasthan, Assam, Nagaland, Gujarat, West Bengal, Jammu and Kashmir, Maharashtra and Sikkim) where the differences are between 10 to 25 points and in the remaining states, the differences are small. This brought out the interstate differentials in IMR and U5MR within the country. However, there are four states where the estimated IMR among the abject poor or moderate poor is lower than that of the non-poor. These states are Haryana, Bihar, Chhattisgarh and Orissa. This is probably due to misreporting of infant deaths as the level of female literacy is low in these states. These states also have higher estimates of IMR among the moderate poor than among the abject poor. There are two more states, namely, Assam and Meghalaya where the estimated IMR among the abject poor is lower by 5 points or more, to those of moderate poor, possibly due to lower sample size. The pattern is similar for the under-five mortality rate. We have not provided the estimated IMR for the abject poor in the states of Himachal Pradesh, Goa and Kerala because the size of the sample is small.

We have provided the estimates of IMR and under-five mortality rate with 95% CI for education, health and wealth of poor in 16 bigger states of India (Table 8 and Table 9). We have not provided the estimates for smaller states of India due to lower sample size and large confidence interval. However, these 16 states constitute more than 90% of India's population and reasonably depict the state differentials in child survival by dimensional poor in India. The differential in IMR by dimensional poor is mixed in states of India.

**Table 8:** Estimated Infant Mortality Rate among dimensional poor in five-year periods preceding the survey in selected states of India, 2005–06.

Sr No	States/India	Infant mortality rate (IMR)			95% CI of estimated IMR		
		Wealth Poor	Educationally Poor	Health Poor	Wealth Poor	Educationally Poor	Health Poor
1	Andhra Pradesh	35	50	41	23–53	33–74	28–58
2	Assam	71	83	74	54–94	59–118	56–99
3	Bihar	65	66	47	52–83	53–81	36–62
4	Chhattisgarh	60	70	74	43–85	49–99	56–99
5	Gujarat	63	63	56	42–95	40–96	41–77
6	Haryana	34	58	36	14–80	36–92	23–56
7	Jharkhand	80	74	74	63–101	57–97	58–95
8	Karnataka	44	49	47	27–70	33–74	34–65
9	Madhya Pradesh	85	76	63	70–104	60–96	51–77
10	Maharashtra	55	44	39	40–75	26–73	28–54
11	Orissa	58	58	50	44–77	40–84	35–71
12	Punjab	85	52	56	44–163	31–88	36–85
13	Rajasthan	76	67	66	57–100	51–88	51–84
14	Tamil Nadu	38	42	39	23–62	20–86	23–63
15	Uttar Pradesh	80	75	78	69–93	65–86	68–89
16	West Bengal	48	50	69	35–66	35–70	53–90
	**India**	**64**	**64**	**56**	**60–68**	**60–68**	**53–60**

**Table 9:** Estimated under-five mortality rate al among dimensional poor in five-year periods preceding the survey in selected states of India, 2000–05.

	States/India	Under five mortality rate (U5MR)			95% CI of estimated U5MR		
		Wealth Poor	Educationally Poor	Health Poor	Wealth Poor	Educationally Poor	Health Poor
1	Andhra Pradesh	71	90	70	58–86	76–107	58–85
2	Assam	110	112	98	95–127	93–135	82–117
3	Bihar	105	95	92	92–119	84–107	80–106
4	Chhattisgarh	105	129	122	88–124	109–151	104–143
5	Gujarat	102	89	84	81–127	71–112	69–101
6	Haryana	55	66	58	34–89	50–88	45–76
7	Jharkhand	115	114	117	100–131	99–132	100–136
8	Karnataka	87	88	73	69–108	73–106	60–89
9	Madhya Pradesh	117	118	109	104–130	104–132	97–123
10	Maharashtra	78	69	58	65–93	55–87	41–71
11	Orissa	105	114	93	91–121	96–136	77–113
12	Punjab	102	56	69	68–151	41–78	52–92
13	Rajasthan	107	100	95	92–125	87–115	82–110
14	Tamil Nadu	51	63	47	38–69	44–89	34–66
15	Uttar Pradesh	128	118	118	118–138	110–127	109–128
16	West Bengal	72	77	89	60–86	64–92	75–106
	**India**	**99**	**95**	**88**	**96–103**	**92–99**	**84–91**

In 9 of the 16 states the estimates of IMR among the educational poor are the same or more than that of wealth poor. These states are Assam, Bihar, Chhattisgarh, Gujarat, Haryana, Orissa, Karnataka, Tamil Nadu and West Bengal. Similarly, there are seven states namely, Andhra Pradesh, Assam, Chhattisgarh, Haryana, Karnataka, Tamil Nadu and West Bengal, where the estimated IMR among the health poor is more than that of the wealth poor. In all other states, the IMR among the wealth poor is higher than that of the educationally poor and health poor. Even in these states, the level of IMR is quite high among the educationally poor or health poor. For few states the confidence interval of dimensional poor varies in large range (for example wealth poor in Punjab).

The pattern in estimated U5MR is similar to that of IMR (Table 9). Among wealth poor, the estimated U5MR varies from 51 in Tamil Nadu to 128 in Uttar Pradesh. Similarly, the estimated U5MR among health poor varies from 47 in Tamil Nadu to 122 in Chhattisgarh.

## Discussion

With the evolution of the human development paradigm [43] and the capability deprivation [44], [45], a shift from money metric poverty to multidimensional poverty has been envisaged in national and international development agenda. However, the measurement and application of multidimensional poverty is limited in many developing countries including India. Though there are concerted efforts to alleviate multidimensional poverty through various developmental schemes like the *National Rural Health Mission (NRHM), the National Rural Employment Guarantee Scheme (NREGS), Sarva Siksha Abhiyan (SSA*), the official estimates of poverty in India are still confined to money-metric poverty, derived from consumption expenditure data.

In this paper, we have attempted to estimate multidimensional poverty in India using the most recent round of National Family and Health Survey data and examined the state of child survival among the abject poor, moderate poor and non-poor households. The choices of indicators are context specific and subject to the availability of data. We have used the most simplified and practical method of deriving dimensional poor; multidimensional poverty is derived using the union approach. We believe that our estimates of abject poor and poor are the minimum by any standard. Our results show that about half of India's population is poor and one-fifth are abject poor (poor in two or all three dimensions) with large rural-urban and inter-state differentials. These estimates are substantially higher compared to the official estimates of poverty for all the states of India. We found that abject poor households had limited access to financial institutions, health insurance schemes and that a higher proportion of abject poor are excluded from the poverty eradication program. The findings of higher poverty among female headed households, large households and households with little or no education (of head of household) are consistent with the findings from other studies. The extent of abject poverty and overall poverty is large in the state of Bihar and least in the state of Kerala. The estimated infant mortality rate and the under-five mortality rate are substantially higher among the abject poor compared to the poor and non-poor across the states.

The estimates of abject poor help us to identify the households suffering from multiple deprivations (poor in two or all three dimensions). These households are unlikely to come out from the poverty trap as they are poor either educationally and economically or economically and in health dimension or in education and health or in all three dimensions. Moreover, they may not be benefitted from various protective measures and the poverty eradication program designed for the poor and marginalized group of population. The measures can address the growing inequality. Hence, the policy prescription is to make special intervention on health care, education and livelihood for those suffering from abject poverty. Within the existing poverty eradication program, we recommend to further classify the abject poor (poorest of the poor) and include them in poverty eradication program. This also calls for special grant and program to backward states of India as the abject poor is concentrated in 12 of the 29 states of India. This will also helps us to reduce the regional inequality in the states.

Further, at national level, we observed that there are no significant differentials in estimates of IMR and under-five mortality rate among the households which were poor in education and wealth dimensions and the differences are small in health and wealth dimension. We have provided some plausible explanation for our results. First the level of health care utilization (such as pre-natal care, natal care and post-natal care, child immunization and child care) are lower among those who are poor in any of the three dimensions. For example, people who are educationally poor might not fully realize the benefits of the maternal and child care while those are economically poor may perceive health services as unaffordable. Second, early marriage of girls and early motherhood, poor nutritional intake of mother during pregnancy (may cause low birth weight), poor environmental condition (unsafe water, no sanitation facilities, use of cooking arrangement, crowding etc), exposure to childhood diseases are equally higher among educationally, economically and health poor. Third, the availability, accessibility and quality of public health services on which people largely relies varied largely among the states of India (very low in the states of Bihar and Uttar Pradesh where the concentration of educational, economic and health poor are more). Fourth, there is some degree of overlapping among the educationally, economically and health poor (as we have seen in abject poverty). Prior studies also documented higher correlation (0.71) of child mortality and child malnutrition while child mortality responds weekly to economic growth [46].

At the state level, there are varying patterns with twelve states having equal or higher estimated IMR among the education poor compared to the wealth poor. Similarly, there are seven states where the estimated IMR among the health poor is higher than that of the wealth poor. This indicates that all these dimensions are equally important in devising strategies to promote child survival and calls for integrating multidimensional poverty in planning and program implementation. The five major cause of child mortality in India, namely, pneumonia, diarrheal diseases, neo-natal infection and birth asphyxia, prematurity and low birth weight and birth trauma that accounts for 62% of all child death [47] are related to poverty. Further, the large differentials in the infant mortality rate and the under-five mortality rate among the abject poor and poor not only validates our measure of multidimensionality poverty but also depicts the poor state of child health in the country. This differential holds well by place of residence and among the states. We acknowledge that this study could not provide the estimates of infant and child mortality for the smaller states in India because the size of the sample was small and limited to the indicators available in the data set.

From policy perspectives, multidimensional poverty clearly demonstrates the multiple deprivation of a household in the key domain of human development, that is, education, health and living standard and inequality in child health outcome. The multidimensional poverty index will serve better for policy formulation as it can address the growing inequality in health care utilization and health outcome among population sub-groups in the country effectively. The small differences in IMR and U5MR among the education poor, wealth poor and health poor demonstrated that the MDGs are interconnected and therefore we need to address these together.

We acknowledge the age sensitive of poverty estimates in education and health domain, due to limitation of data. Those households without children in the age group 6–14 years will be recorded as non-poor in this variable. However, all household had an adult member and so represent the adult literacy component. With respect to health domain, healths of children in 6–14 years and adults 50 years and above are not covered in the survey. But 83% household in India had a women in 15–49 age group and 37% households had a child under five year age group may reasonably capture the health domain.

### Research and Policy Implications

The implications of this study are both for research and policy. With respect to research, the paper demonstrated the robust measurement of multidimensional poverty and its linkages with child survival using data from a large scale population based survey. The selection of indicators is illustrative and contextual. We begin the work by estimating the poverty and differentials in child death by poverty in India and suggest carrying out more research on health care differentials and linkages of poverty and health. We recommend to exploit the richness of secondary data collected in various population based surveys including (but not limited to) the Demographic and Health Surveys (DHS) of many developing countries and develop the measurement of multidimensional poverty at national and sub-national levels, using context specific variables. The multidimensional poverty index used uniform variables across the countries and more of comparative analyses. It will be useful to link multidimensional poverty with process and outcome indicators such as health care utilization, health and health inequality in the population and derive useful inferences for evidence based planning.

Based on the findings, the foremost policy implication from the study is moving from the long contested measure of consumption poverty to multidimensional poverty in planning and program implementation of the centre and state governments, by developmental agencies and various organizations. The Planning Commission, Govt. of India, has recognized the multidimensional nature of poverty and we suggest working in this direction so as to arrive at more precise estimates of poverty. This measure may end the recent debate of poverty line cut-off of 32 rupees in urban and 26 rupees in rural area that received sharp criticism from various corner. Second, the exclusion of a high proportion of the abject poor in BPL programs which are specifically designed for conditional cash transfer and eradicating extreme poverty is a serious concern. That only two-fifths of abject poor households had a BPL card is an indication that majority of the poor are excluded from the poverty eradication program. Hence, the inclusion criterion and the transparency in the allocation of BPL cards need to be examined so as to reduce poverty. Third, we suggest in using multidimensional poverty as one of the criteria in the transfer of fiscal resources from the centre to the state. Among other factors, the 13^th^ Finance Commission recommended deprivation and percentage of Scheduled Castes and Tribes in rural areas (based on 2001 census) as criteria in the transfer of central funds to the states. This needs a collective effort and consensus among the states of India to fight against poverty and hunger in line with the commitment of developing and developed countries in realizing the MDGs. Last, we recommend targeted intervention in access to health care, education and livelihood for the abject poor irrespective of caste, creed, religion and space so as to address the equity lens and realize the MDGs.

## Supporting Information

Appendix S1 **Unweighted sample size, India, 2005–06.**
(DOCX)

Appendix S2 **Confidence Interval of estimated IMR and U5MR by abject poor, moderate poor and non-poor in states of India.**
(DOCX)

## References

[1] Wang L (2003). Determinants of child mortality in LDCs: Empirical findings from demographic and health surveys.. Health Policy.

[2] Gwatkin DR (2005). How much would poor people from faster progress towards the millennium development goals for health?. Lancet.

[3] Alkire S, Foster J (2011). Understanding and Misunderstandings of Multidimensional Poverty Measurement..

[4] Alkire S, Foster J (2009).

[5] Alkire S (2007). The missing dimensions of poverty data: Introduction to the special issue.. Oxford Development Studies.

[6] UNDP (1997). Human Development Report..

[7] Bourguignon F, Chakravarty SR (2003). The measurement of multidimensional poverty.. Journal of Economic Inequality.

[8] Gordon D, Namdy S, Pantazis C, Pemberton S, Townsend P (2003). The distribution of child poverty in the developing world..

[9] Wagle U (2008). Multidimensional poverty: An alternative measurement approach for the United states?. Social Science Research.

[10] Calvo C (2008). Vulnerability to Multidimensional Poverty: Peru, 1998-2002.. World Development.

[11] Klasen S (2000). Measuring poverty and deprivation in South Africa.. Review of Income and Wealt.

[12] Sahn DE, Stifel DC (2000). Poverty comparison over-time and across countries in Africa.. World Development.

[13] Booysen F, Von Maltitz M, Du Rand G (2008). Using an asset index to assess trends in poverty in seven Sub-Saharan African countries.. World Development.

[14] Srinivasan K, Mohanty SK (2008). Household deprivation and its linkages with reproductive and child health care and health outcome.. Economic Political Weekly.

[15] Akire S, Santosh M (2010). Acute Multidimensional Poverty: A New index for developing countries..

[16] UNDP (2010). Human Development Report 2010..

[17] Planning Commission, Government of India (2007). http://planningcommission.nic.in/news/prmar07.pdf.

[18] Planning Commission, Government of India (2009). Report of the expert group to review the methodology for estimation of poverty..

[19] Sundaram K (2003). On identification of households below poverty line: some comments on the proposed methodology.. Economic and Political Weekly.

[20] Ram F, Mohanty SK, Ram U (2009). Understanding the distribution of BPL cards: All-India and selected states.. Economic Political Weekly.

[21] Alkire S, Seth S (2008a). Multidimensional Poverty and BPL measures in India: A comparison of methods..

[22] Alkire S, Seth S (2008b). Determining BPL status: Some Methodological Improvements.. Indian Journal of Human Development.

[23] International Institute for Population Sciences (IIPS) and Macro International (2007). National Family Health Survey (NFHS 3), 2005-06, India: Volume I..

[24] Mohanty SK, Pathak PK (2009). Rich-poor gap in utilization of reproductive and child health care services in India, 1992-2005.. Journal of Biosocial Sciences.

[25] Kanjilal B, Mazumdar PG, Mukherjee M (2010). Nutritional status of children in India: household socio-economic as the contextual determinant.. International Journal for equity in Health.

[26] Kumar A, Mohanty SK (2011). Intra-urban differentials in the utilization of reproductive health care in India, 1992-2006.. Journal of Urban Health.

[27] Paul VK, Sachdev HS, Mavalankar D (2011). Reproductive health, and child health and nutrition in India: meeting the challenges.. Lancet.

[28] Rice AL, Sacco L, Hyder A, Black RE (2000). Malnutrition as an underlying cause of childhood deaths associated with infectious disease in developing countries.. Bulletin of the World Health Organsiation.

[29] Caulfield LE, de Onis M, Blossner M, Black RE (2004). Undernutrition as an underlying cause of child deaths associated with diarrhea, pneumonia, malaria, and measles.. American Journal of Clinical Nutrition.

[30] Grantham-McGregor S, Cheung YB, Cueto S (2007). Developmental potential in the first 5 years for children in developing countries.. Lancet.

[31] Black RE, Allen LH, Bhutta ZA, de Onis M (2008). Maternal and child undernutrition: global and regional exposures and health consequences.. Lancet.

[32] Barbin BJ, Hakimi M, Pelletier D (2001). Iron-Deficiency Anemia: Reexamining the Nature and magnitude of the Public Health Problem.. The Journal of Nutrition..

[33] Campbell Oona MR, Graham WJ (2006). Strategies for reducing maternal mortality: getting with what works.. Lancet.

[34] Rutstein S, Johnson K (2004). The DHS Wealth Index in DHS Comparative Reports..

[35] Filmer D, Pritchett LH (2001). Estimating wealth effects without expenditure data- or tears: An application to educational enrolments in states of India.. Demography.

[36] Mohanty SK (2009). Alternate wealth index and health estimates in India.. Genus.

[37] Deutsch J, Silber J (2005). Measuring multidimensional poverty: An empirical comparison of various approaches.. Review of Income and Wealth..

[38] Planning Commission, Government of India (2002). National Human Development Report..

[39] Rutstein S (2000). Factors associated with trends in infant and child mortality in developing countries during the 1990s.. Bulletin of the World Health Organization..

[40] Hatt LE, Waters HR (2006). Determinants of child mortality in Latin America: a pooled analysis of interactions between parental education and economic status.. Social Science Medicine.

[41] Office of the Registrar General of India (RGI) and Centre for Global Health Research (CGHR) (2009). Report on Cause of Death in India, 2001-2003..

[42] You D, Wardlaw T, Salama P, Jones G (2010). Levels and trends in under-5 mortality, 1990-2008.. The Lancet..

[43] UNDP (1990). Human Development Report..

[44] Sen AK (1992). Inequality reexamined..

[45] Sen AK (1999). Development as freedom..

[46] Bhat PNM, Zavier F (1999). Findings of national Family Health survey: Regional Analysis, Economic and Political Weekly..

[47] The Million Death Study Collaborators (2010). Cause of neonatal and child mortality in India: a nationally representative mortality survey.. The Lancet..

