# Supplementary material for: Multidimensional Poverty and Child Survival in India
Source: PLoS One. 2011 Oct 27;6(10):e26857. doi: 10.1371/journal.pone.0026857 (PMC3203176; doi:10.1371/journal.pone.0026857)
Supplement: Appendix S1 — Unweighted sample size, India, 2005–06. (DOCX) [file pone.0026857.s001.docx]

**Appendix S1: Unweighted sample size, India, 2005-06**

| Households/ Women | Combined | Rural | Urban |
| --- | --- | --- | --- |
| Number of Households | 1,09,041 | 58,805 | 50,236 |
| Number of households with at least one women aged 15-59 | 90,014 | 48,927 | 41,087 |
| Number of households with at least one child aged 0-59 months | 40,593 | 23,961 | 16632 |
| Number of households with at least one child aged 7-14 years | 53,230 | 31,121 | 22,019 |
| Number of women interviewed | 124,385 | 67,424 | 56961 |
