# Supplementary material for: Multidimensional Poverty and Child Survival in India
Source: PLoS One. 2011 Oct 27;6(10):e26857. doi: 10.1371/journal.pone.0026857 (PMC3203176; doi:10.1371/journal.pone.0026857)
Supplement: Appendix S2 — Confidence Interval of estimated IMR and U5MR by abject poor, moderate poor and non-poor in states of India. (DOCX) [file pone.0026857.s002.docx]

**Appendix S2 Confidence Interval of estimated IMR and U5MR by abject poor, moderate poor and non- poor in states of India**

|  | India/States | 95% CI of IMR | | | | 95% CI of U5MR | | | |
| --- | --- | --- | --- | --- | --- | --- | --- | --- | --- |
| Sr No |  | Abject poor | Moderate poor | Non- poor | All | Abject poor | Moderate poor | Non- poor | All |
| 1 | Jharkhand | 65-105 | 49-94 | 33-63 | 56-81 | 91-112 | 78-115 | 52-89 | 91-112 |
| 2 | Uttar Pradesh | 70-195 | 64-84 | 56-77 | 67-80 | 120-140 | 94-111 | 78-94 | 100-110 |
| 3 | Tripura | 45-138 | 27-90 | 21-70 | 36-72 |  | 39-86 | 29-62 | 58-80 |
| 4 | Madhya Pradesh | 63-97 | 51-81 | 36-65 | 56-74 | 86-93 | 81-106 | 53-74 | 86-101 |
| 5 | Arunachal Pradesh | 49-110 | 43-98 | 26-92 | 49-83 | 78-128 | 80-127 | 49-98 | 81-109 |
| 6 | Rajasthan | 55-96 | 54-91 | 39-79 | 57-79 | 85-117 | 89-120 | 58-88 | 84-102 |
| 7 | Mizoram | 29-158 | 28-89 | 13-40 | 24-49 | 89-199 | 97-106 | 80-116 | 93-124 |
| 8 | Assam | 48-95 | 57-101 | 34-77 | 54-80 | 79-99 | 69-101 | 50-82 | 79-99 |
| 9 | Manipur | 40-106 | 26-59 | 14-32 | 25-41 | 55-101 | 42-70 | 30-48 | 42-56 |
| 10 | Delhi | 38-112 | 35-83 | 22-50 | 34-58 | 47-100 | 57-97 | 28-47 | 44-62 |
| 11 | Punjab | 33-115 | 40-88 | 22-48 | 34-57 | 40-99 | 59-95 | 32-52 | 46-64 |
| 12 | Chhattisgarh | 42-89 | 60-103 | 57-103 | 61-88 | 90-110 | 79-112 | 66-98 | 90-110 |
| 13 | Uttaranchal | 34-111 | 36-82 | 18-47 | 32-57 | 63-121 | 72-112 | 42-66 | 60-80 |
| 14 | Jammu and Kashmir | 32-111 | 29-69 | 29-61 | 35-58 | 46-109 | 43-72 | 36-60 | 44-62 |
| 15 | Gujarat | 37-91 | 46-87 | 24-55 | 41-64 | 47-86 | 62-93 | 51-79 | 67-86 |
| 16 | Bihar | 45-73 | 50-89 | 48-94 | 54-74 | 88-113 | 69-99 | 61-95 | 82-99 |
| 17 | Sikkim | 18-158 | 17-64 | 18-59 | 23-53 | 54-147 | 90-142 | 95-142 | 97-130 |
| 18 | Nagaland | 33-83 | 35-67 | 23-46 | 33-51 | 72-119 | 57-84 | 46-67 | 39-75 |
| 19 | Orissa | 36-74 | 44-86 | 55-98 | 52-75 | 80-99 | 64-96 | 59-89 | 80-99 |
| 20 | West Bengal | 36-72 | 45-79 | 27-52 | 40-58 | 54-68 | 55-81 | 35-53 | 54-68 |
| 21 | Karnataka | 27-73 | 39-71 | 28-52 | 37-54 | 60-74 | 65-91 | 43-62 | 60-74 |
| 22 | Maharashtra | 28-72 | 36-62 | 25-43 | 32-46 | 47-58 | 50-70 | 35-49 | 47-58 |
| 23 | Meghalaya | 26-73 | 34-78 | 22-68 | 34-60 |  | 53-89 | 32-67 | 51-78 |
| 24 | Haryana | 22-81 | 26-61 | 32-69 | 33-57 | 35-80 | 48-80 | 39-65 | 47-65 |
| 25 | Andhra Pradesh | 26-65 | 31-60 | 29-54 | 33-50 | 55-68 | 57-68 | 37-56 | 55-68 |
| 26 | Tamil Nadu | 12-68 | 28-61 | 17-38 | 24-41 | 39-53 | 44-71 | 31-39 | 39-53 |
| 27 | Himachal Pradesh | ** | 22-69 | 20-48 | 23-45 | 14-125 | 37-76 | 27-48 | 33-51 |
| 28 | Goa | ** | 7-47 | 8-28 | 8-25 | 23-139 | 31-72 | 15-32 | 23-39 |
| 29 | Kerala | ** | 5-44 | 8-26 | 9-25 | 15-27 | 9-38 | 14-28 | 15-27 |
|  | * Not estimated |  |  |  |  |  |  |  |  |
